# Cell-Type-Specific Differentiation and Molecular Profiles in Skin Transplantation: Implication of Medical Approach for Genetic Skin Diseases

**DOI:** 10.1155/2011/501857

**Published:** 2011-11-17

**Authors:** Noritaka Oyama, Fumio Kaneko

**Affiliations:** ^1^Institute of Dermato-Immunology and Allergy, Southern Tohoku Research Institute for Neuroscience, Koriyama, Fukushima 963-8563, Japan; ^2^Institute of Dermato-Immunology and Allergy, Research Institute for Neuroscience, Southern Tohoku General Hospital, 7-115 Yatsuyamada, Koriyama, Fukushima 963-8563, Japan

## Abstract

Skin is highly accessible and valuable organ, which holds promise to accelerate the understanding of future medical innovation in association with skin transplantation, engineering, and wound healing. In skin transplantation biology, multistage and multifocal damages occur in both grafted donor and perilesional host skin and need to be repaired properly for the engraftment and maintenance of characteristic skin architecture. These local events are more unlikely to be regulated by the host immunity, because human skin transplantation has accomplished the donor skin engraftment onto the immunocompromised or immunosuppressive animals. Recent studies have emerged the importance of *α*-smooth muscle actin- (SMA-) positive myofibroblasts, via stage- and cell-specific contribution of TGF*β*, PDGF, ET-1, CCN-2 signalling pathways, and mastocyte-derived mediators (e.g., histamine and tryptase), for the functional reorganisation of the grafted skin. Moreover, particular cell lineages from bone marrow (BM) cells have been shown to harbour the diferentiation capacity into multiple skin cell phenotypes, including epidermal keratinocytes and dermal endothelial cells and pericytes, undercontrolled by chemokines or cytokines. From a dermatological viewpoint, we review the recent update of cell-type- and molecular-specific action associated with reconstitution of the grafted skin and also focus on the novel application of BM transplantation medicine in genetic skin diseases.

## 1. Introduction

Skin represents a substantial part of mammalian ectoderm, which is normally exposed by various exogenous stimuli, for example, UV irradiation, infection, temperature, moisture, and mechanical aspects [1]. Because skin is highly accessible to any of diagnostic and treatment procedures, studies for skin transplantation, as well as skin engineering and wound healing, hold great promise to accelerate the understanding of underlying pathophysiology for future medical innovation utilizing self-made or more feasible skin equivalents. Over a thousand gene-mutant loci for inherited human disorders have been reported thus far [2], and approximately one-third of these have been known to exhibit the relevant skin abnormalities, in which gene targeting therapy has yet to be standardised practically. Skin transplantation strategy and its relevant technology may thus remain highly safely and benefitly, in such skin conditions, as well as skin plastic surgery. 

Pathologically, the predominant cell populations in mammalian skin comprise dermal fibroblasts and epidermal keratinocytes, both of which show different morphology and function. Ours and other studies have utilized two-/three-dimensional coculture or complex “organotopic” systems, allowing to evaluate the importance of paracrine interaction between the two different cell types [3, 4]. Upon these *in vitro* skin equivalent assays, our potential interests persist to know how these two cell types are reorganised properly in the grafted skin. At the site of the skin graft, multistage and multifocal damages of the donor/host skin, microhaemorrhage, and later excess fibrosis in the dermis might have eventually occurred, and the grafted skin, therefore, needs to be repaired and reconstituted through these inevitable events. More specifically, little is known about how the biological architecture characteristic for the skin (e.g., stratified squamoid epithelia and dermis intermixed with extracellular matrices) can be maintained after the skin transplantation. One may consider that a sort of particular cell phenotypes plays a central role in the orchestration of the skin reconstitution, and, if so, under what particular circumstances for this process? The chain of these biological events is more unlikely to be regulated by cellular and humoral immunity in the host, because vast majority of *in vivo* researches for human skin transplantation has accomplished the donor skin engraftment onto the immunocompromised animals, such as nude and athymic mice, or those treated with immunosuppressive agents or the particular subset (CD4^+^ CD25^+^) of T cells [5, 6]. Thus, a somewhat study limitation may often enable us to access to the insight associated with the skin transplantation immunobiology.

For understanding the cell-specific action in the skin transplantation, evidences from bone marrow (BM) transplantation study may in part bring the clue. Native BM cells comprise the substantial proportion of cell sources that have roles in tissue homeostasis, repair, and regeneration. These cell populations are originated from either haematopoietic or mesenchymal stem cells and subpopulations that are capable of differentiating into multiple cell lineages [7, 8]. A series of recent research progress have emerged that BM cells can provide not only fibroblastic cells but also epithelial cells in the lung and intestinal epithelium and skin [9]. Particularly in skin, a transplantation of sex (XY) chromosome-mismatched BM cells or intrinsically labelled BM cells has demonstrated that keratinocyte-specific marker-positive BM cells appeared in the epidermis, hair follicles and sebaceous glands [10–15]. Moreover, in patients who underwent a BM transplantation, donor BM cells displaying wide-ranged keratinocyte markers (pan-keratin) were detectable in the epidermis and maintained for over 3 years after the transplantation [16]. These data series suggest that the transdifferentiated keratinocytes from BM cells not only aid the impairment of the residual epidermal function after transplantation but also participate in the compensation of the epidermal circumstances at the affected skin sites. On this basis, the BM-derived keratinocyte populations are secured functionally and structurally as a baseline stable supply. However, it remains unclear (i) how the BM cells are recruited strictly into the grafted skin, and, if once they failed this process, how it can be corrected properly, (ii) how the recruited BM cells contribute functionally to the local skin regeneration, and, more interestingly, (iii) whether the newly established epithelial-mesenchymal interaction can maintain the local skin homeostasis analogous to the host skin. From a dermatological view point, this paper focuses mainly on these attractive points in association with the cell-type-specific reorganization in the skin transplantation, as well as the relevant molecular profiles. These advanced evidences will help to ask how we can establish the better medical approaches for persistent skin wound condition, particularly in genetic skin diseases.

## 2. Myofibroblasts in Skin Transplantation: What It Is, How It Acts, and Where It Comes from?

After skin transplantation, the grafted skin sites need to repair some inevitable minor trauma, for example, occasional haemorrhage caused by microvascular damage, later excess microfibrosis, or even focal necrotic changes, in order to adapt to the host skin circumstance. These minor tissue damages in the grafted donor skin and/or perilesional host skin may primarily drive the recruitment of the particular subset of fibroblastic cells, termed “myofibroblasts” that specifically express the intracellular structural protein *α*-smooth muscle actin (*α*-SMA) [17]. Myofibroblasts can migrate into the grafted skin and subsequently produce collagens, fibronectin, and proteoglycans to reconstitute the local extracellular matrix (ECM) network in the dermis [18, 19]. During this process, *α*-SMA is reorganised into the complexes of stress fibre for biological connecting to the surrounding ECM molecules and participates in the exert contraction and mechanical tension, as well as reconstitution of primary intra-/intercellular skeleton, for the establishment of the functional remodelling of connective tissue. In contrast, persistence and/or aberrant increase of myofibroblast action may be responsible for fibrosclerotic skin diseases, such as scleroderma or morphea [20]. Another *in vitro* observation with human embryonic stem (hES) cells utilizing a three-dimensional skin model has shown that hES cell-derived mesenchymal cells that constitutively express *α*-SMA can promote multilayered epithelium and the resultant wound healing, with increased production of hepatocyte growth factor HGF, an essential factor for skin development and repair [21, 22]. This characteristic cell phenotype might also be analogous to myofibroblastic cell lineage, with possible implication of epidermal-mesenchymal crosstalk in a HGF-dependent manner. 

**Figure 1:**
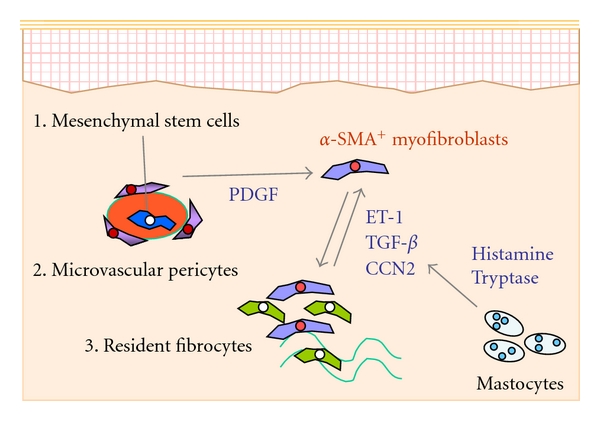
Schematic model of myofibroblast differentiation in the skin. The local myofibroblasts characteristic for *α*-SMA expression are originated from multiple cell sources in the skin and nominated from at least 3 distinct cell sources: BM-derived mesenchymal stem cells, microvascular pericytes, and resident fibroblasts in the donor skin graft and/or in the perilesional host skin. Some molecules can organise the cell-type-specific differentiation into dermal myofibroblasts.

The local myofibroblasts—without regard to the newly recruited into the donor grafted skin or locally residential cells—are considered to be originated from multiple cell sources *in vivo*. Current concept favours at least 3 distinct cell sources of skin myofibroblasts (Figure 1): (i) pericytes that composed of skin microvasculature, (ii) resident fibroblasts in the donor grafted skin and/or in the perilesional host skin, or (iii) BM-derived mesenchymal stem cells [17]. These myofibroblast sources may be selective and changeable appropriately in a skin-damage-dependent manner [23, 24]. However, there have been no convincing data for what percentage of the particular cell-lineage-derived myofibroblasts is involved in the reconstitution of the skin graft. Also, little is known about whether any biological thresholds of the myofibroblast recruitment exist in this event.

## 3. Molecular Contribution for Myofibroblast Differentiation

Extensive reviews on TGF*β*, PDGF, ET-1, CCN-2 signalling, and several mediators from mastocytes and the potential contribution of this pathway in the myofibroblast biology have been reported elsewhere [20, 25, 26] (Figure 1). Each of these supports a variety of biological action in skin fibroblasts and is most likely to make a complex interrelationship to promote the skin wound repair, remodelling, and reorganization after the skin transplantation.

Five TGF*β* isoforms, TGF*β*1–5, exist in mammals and are generated initially as biologically latent precursors, enabling them to bind to a heteromeric receptor complex (a type I and II receptor complex). The former receptor phosphorylates Smad2 and 3, which subsequently binds to Smad4, and finally activates gene transcription in fibroblasts. Activation of the TGF*β* signalling causes increased production of collagen I and ECM molecules [27, 28], as well as myofibroblast differentiation and *α*-SMA expression, in parallel with CCN expression and *α*-SMA-dependent stress fibre formation [29–32].

There are 3 isoforms of endothelin, ET-1, 2, and 3 [33]. ET-1 is the major isoform in human and is produced by various cell types, including endothelial cells, BM cells, haematopoietic cells, cardiomyocytes, and fibroblasts. ET-1 is secreted as a 212-amino acid precursor (prepro-ET-1) and enzymatically cleaved to a biologically active 21-amino acid peptide, which can bind to the two distinct receptors (ET-A and ET-B). ET-1 induces—in cooperation with TGF-*β* pathway—myofibroblast formation and migration and ECM contraction via binding to ET-A/B receptors and the resultant activation of downstream signalling molecules, Akt/rac [34, 35].

CCN2, a member of the CCN family of matricellular proteins, is induced by TGF*β* and ET-1 system, vice versa, and is, therefore, considered an essential cofactor required for the particular subsets of TGF*β* cascade, FAK/Akt/PIP3K [33, 36]. CCN2 can activate the fibrotic phenotype of cells and also support a variety of biological TGF*β* action, such as type I collagen synthesis, *α*-SMA expression, and promotion of cell-ECM interaction [37, 38].

Platelet-derived growth factor (PDGF) family members include PDGF-AA, -AB, -BB, -CC, and -DD, and bind to two different PDGF receptors *α* and *β* [39]. PDGF can enhance multiple cell types, including neutrophils, macrophages, fibroblasts, and smooth muscle cells, to proliferate and migrate them into the skin wound, and also stimulate the differentiation into myofibroblasts, thus contributing to the local skin remodelling and contracture [40]. Mice treated with imatinib mesylate, a PDGF*β* receptor-specific tyrosine kinase inhibitor, exhibited delayed skin wound healing with decreased levels of the local myofibroblast number, collagen type I expression [41], and noncanonical TGF*β* signal network [42, 43], suggesting the direct biological action of PDGF in skin regeneration. Also, a recent study has suggested the potential contribution of a subset of PDGFRa-positive BM cell population in the epidermal keratinocyte differentiation and reorganization in mice skin [23].

Mastocytes have pleiotropic action for fibroblast biology by secreting a variety of chemical mediators and cytokines. In cell co-culture and skin-equivalent culture systems, for example, human mastocyte line HMC-1 cells can induce the expression of *α*-SMA in fibroblasts, via paracrine action of histamine and a serine protease tryptase, thereby contributing to the fibroblast-dependent skin contraction [44].

## 4. Mesenchymal Stem Cells in Skin Transplantation

### 4.1. Keratinocyte Differentiation

**Figure 2:**
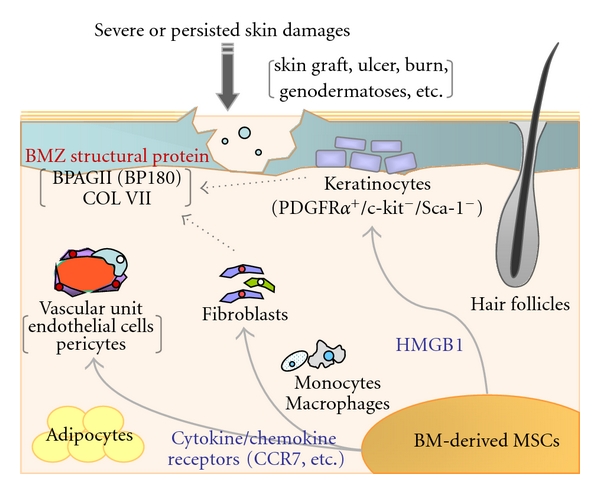
Transdifferentiation of bone-marrow-derived mesenchymal stem cells (MSCs) into the multiple skin component cells. The particular subset(s) of allogenically transferred MSCs, a PDGFR*α* ^+^/c-kit^−^/Sca-1^−^ lineage, can differentiate into the keratin-marker-positive epidermal keratinocytes via a paracrine action of HMGB1. In another cascade, the transdifferentiation activity of the MSCs into other skin components such as vasculature (endothelial cells and pericytes) and dermal fibroblasts—albeit much lesser with monocytes, macrophages, and adipocytes—is accelerated by certain cytokine/chemokine signalling, especially CCR7-SLC/CCL21 pathway. These BM-derived multiple cell lineages can be a potential source for supplying skin structural molecules, such as type VII collagen (COLVII) and type XVII collagen (BPAGII; BP180), both of which are essential anchoring molecules in the basement membrane zone (BMZ).

Accumulating evidence has gained the possibility that mesenchymal stem cells (MSCs) can contribute to the skin wound repair and development. For example, infusion of genetically engineered green fluorescent protein (GFP-) expressing BM cells into mice utero results in accumulation of a certain subpopulation of GFP-positive cells in nonwounded skin dermis, particularly in high association with hair follicles [45]. More precisely, *in vivo* transplantation of sex (XY) chromosome-mismatched human BM cells or GFP-expressing murine BM cells has demonstrated that, at least by 4 weeks after the transplantation, keratinocyte-marker-positive BM cells appeared in the epidermis, hair follicles, and sebaceous glands [10–15], sites that harbour skin stem cell niches [46] (Figure 2). Thereafter, the locally recruited BM cells into the grafted skin in mice can be maintained at least for 5 months [23]. Considering the short turnover time of mice skin (2-3 weeks), the long-residing BM-derived epithelial cells are most likely to contain subpopulation(s) of epithelial progenitor/stem cells. This characteristic cell population constitutively expresses PDGF receptor- (PDGFR-) *α*, but neither c-kit nor Sca-1 [*23*], and the differentiation activity is accelerated by a paracrine action of heparin-binding molecules from the skin graft, especially high-mobility group box 1 (HMGB 1) [47] (Figure 2).

However, mice and human BM transplantation studies have revealed that BM-derived keratinocytes account for an extremely rare population in both wounded and non-wounded skin epidermis, for example, almost undetectable levels or only less than 0.0003% of all keratinocytes in the mice epidermis [24] and 0.14% of those in human epidermis [15]. These poor cell numbers are in agreement with the baseline observation of recent reports, and, in parallel, they never aggregate in the epidermis but mostly present therein as a single cell [15, 23]. Conceptionally, the relatively scarcity of such cells may, therefore, raise questions about their biological significance in the skin engraftment. Besides, the recruited BM cells can be a potential source for supplying skin structural molecules, such as type VII (COL7) and type XVII collagens (BP180), both of which are essential anchoring molecules in dermal-epidermal junction (Figure 2). Loss-of-function mutations of these genes cause subtypes of genetic skin fragility and scarring diseases, recessive dystrophic (RDEB, OMIM no. 226600), and junctional epidermolysis bullosa (non-Herlitz JEB, OMIM no. 226650), respectively. Embryonic and postnatal transplantation of BM cells into mice lacking type VII or XVII collagens can successfully ameliorate the persisted skin wound and fragility by newly generation of the defected skin molecules [45]. Most convincing evidence from a clinical trial of allogeneic whole BM transplantation in a patient with RDEB has successfully shown that BM cells can repair the skin wound and restore the defected COL7 expression in the skin basement membrane zone [16]. Overall, these data suggest that minimally transdifferentiated BM cells are indeed sufficient for the generation of deficient skin protein(s) and restore the fragile skin condition.

### 4.2. Differentiation of BM Cells into Multiple Skin Cells

Along with a streamline for the functional epidermal differentiation of BM cells, a most recent investigation has explored that BM-derived MSCs intravenously injected can differentiate into multiple skin cell lineages, including epidermal keratinocytes, and dermal endothelial cells and pericytes, finally contributing to skin wound repair in mice, suggesting upregulation of angiogenic properties in the host skin [15] (Figure 2). This MSC phenotype harbours several chemokine receptors, especially CCR7, a receptor of SLC/CCL21 that enables MSCs to migrate into the local tissues [48, 49]. Perilesional skin injection of SLC/CCL21, but not thymus and activation-regulated chemokine (TARC), can increase the baseline differentiation of MSCs into the wound skin and close the wound. In this study, the trans-differentiation activity of bulk MSCs into multiple skin cell phenotypes seems higher comparative with previous reports: ~0.14% of GFP-positive MSCs into epidermal keratinocytes, ~13.2% into endothelial cells, and ~33.0% into pericytes in the dermis, albeit much lesser with monocyte/macrophage and adipocyte lineages [23]. Interestingly, the recruitment of BM-derived cells is significant in the grafted skin and long-standing damaged skin, including RDEB [45, 50], whereas it is much lesser or almost negligible level in most of transiently established skin wound healing models [23, 24]. The proportion of the recruited and/or transdifferentiated BM cells seems considerably variable by the skin damage and its period.

## 5. Future Perspective

Despite the recent dramatic progress in the skin transplantation and wound healing studies, we now face some inconclusive debates that need to be addressed in future; how much of the trans-differentiation activity of BM-derived MSCs is indeed influenced by differences in individuals, for example, age, medical history and ongoing treatments, and affected skin sites. Are there any biological thresholds to recruit MSCs or to induce *α*-SMA^+^ myofibroblasts for the proper skin engraftment and wound healing; if any, how can we analyse them quantitatively? Which soluble molecules or combination of these (e.g., SLC/CCL21, HMGB1, and PDGF; Figure 2)—if we add exogenously—are more efficient to ensure the favourable outcome of the transplantation events? Particularly in allogenic BM transfer, do these supplemental additives affect the baseline incidence of life-threatening complications, such as GvHD? These parameters should be estimated precisely, but instead the study limitation may include the restricted category of the targeted skin diseases, like lack-of-functional protein genodermatoses, and, therefore, the difficulty that most of advanced results comes from researches associated with skin wound healing.

## 6. Summary

Skin transplantation researches have gradually been saturated by multiangle evidence and interpretation from the relevant organ transplantation and provide multiple therapeutic implications. BM-derived cells with pluripotent differentiation capacity into multiple skin components may serve as target and/or vector cells for innovative gene therapy and proper reconstitution of various wounded skin, particularly in genetic skin diseases.

##  Conflict of Interests

The authors declared that there is no conflict of interest.
